# Distractor Efficiency in an Item Pool for a Statistics Classroom Exam: Assessing Its Relation With Item Cognitive Level Classified According to Bloom’s Taxonomy

**DOI:** 10.3389/fpsyg.2018.01585

**Published:** 2018-08-28

**Authors:** Silvia Testa, Anna Toscano, Rosalba Rosato

**Affiliations:** ^1^Department of Psychology, University of Turin, Turin, Italy; ^2^UMR INSERM 1246, SPHERE, Methods in Patient-Centered Outcomes and Health Research, University of Nantes, University of Tours, Nantes, France

**Keywords:** distractors, Bloom’s taxonomy, multiple-choice items, item analysis, Rasch model

## Abstract

Multiple-choice items are one of the most commonly used tools for evaluating students’ knowledge and skills. A key aspect of this type of assessment is the presence of functioning distractors, i.e., incorrect alternatives intended to be plausible for students with lower achievement. To our knowledge, no work has investigated the relationship between distractor performance and the complexity of the cognitive task required to give the correct answer. The aim of this study was to investigate this relation, employing the first three levels of Bloom’s taxonomy (Knowledge, Comprehension, and Application). Specifically, it was hypothesized that items classified into a higher level of Bloom’s classification would show a greater number of functioning distractors. The study involved 174 items administered to a sample of 848 undergraduate psychology students during their statistics exam. Each student received 30 items randomly selected from the 174-item pool. The bivariate results mainly supported the authors’ hypothesis: the highest percentage of functioning distractors was observed among the items classified into the Application category (η^2^ = 0.024 and Phi = 0.25 for the dichotomized measure). When the analysis controlled for other item features, it lost statistical significance, partly because of the confounding effect of item difficulty.

## Introduction

Currently, the use of standardized and computerized tests for learning evaluation is an interesting and relevant topic for those involved in the learning process, evaluation and instruction. As far as student assessment is concerned, it is often possible to assemble a pool of multiple choice elements (MCIs) to be administered during an exam. Given its advantage in reducing testing time, this form of evaluation has become popular and is frequently used in very large university classes (DiBattista and Kurzawa, 2011). In addition, MCIs can be used during university exams to accurately assess students by administering questionnaires that require different cognitive skills to obtain a correct answer (Coppedge and Hanna, 1971; Matlock-Hetzel, 1997). Through the MCIs, teachers can assess different student capacities, such as knowledge, skills, and specific academic abilities (Haladyna et al., 2002). With the MCIs, factual knowledge and more complex capabilities can be investigated, such as making inferences, solving problems, organizing information, or integrating the ideas and concepts of a topic (Hancock, 1994). The most widely used classification of cognitive processes is Bloom’s taxonomy (Bloom, 1956; Kim et al., 2012; Omar et al., 2012; Yahya et al., 2013).

### Bloom’s Taxonomy

Bloom’s taxonomy is a tool that can be used to classify the levels of reasoning skills required in classroom situations. It consists of the following six levels: *Knowledge*, *Comprehension*, *Application*, *Analysis*, *Synthesis*, and *Evaluation*.

*Knowledge* is the student’s ability to remember the information stored during the learning process. Studying the concepts, rules and definitions presented in textbooks helps to correctly answer the questions that belong in this category. *Comprehension* is the ability to demonstrate understanding of the information presented in the items. Skills such as the translation from one type of representation to another, interpretation, and classification are necessary to provide the right answer to the questions that belong to this category. *Application* refers to problem-solving skills. Students who answer such questions must apply learned information and concepts in new and concrete situations. *Analysis* is the mastery of organizing or dividing a whole into its component parts. People with an overview of the individual elements can draw conclusions or know how to make inferences based on some cues. At this level, organizing individual parts with principles and criteria is also a desired skill. *Synthesis* is the ability to combine elements or ideas to form something new and unique such as a project, a proposal or a product. Synthesis is best assessed by essay format, by which the examinee must demonstrate all the learned skills (Aviles, 2000). The last level of cognitive processing is *Evaluation*, which is a competence in making judgments about methods using internal or external principles (Omar et al., 2012).

### Item and Distractor Quality

The performance of achievement items is typically assessed in terms of difficulty and discrimination power.

Depending on the theoretical approach, difficulty is assessed differently and is defined as the percentage of correct answers (*P*-value) in the Classical Test Theory (CTT) approach and as the skill level required to have a 50% chance of giving the correct answer in the Rasch modeling approach (De Ayala, 2013). Discrimination power refers to the ability to distinguish between high and low achievers. The right answer must have a positive discrimination (Tarrant et al., 2009; DiBattista and Kurzawa, 2011).

When the test consists of MCIs, the performance of distractors must also be considered: implausible options lengthen the duration of the test without improving the accuracy of the assessments (DiBattista and Kurzawa, 2011). The quality of the distractor can be evaluated by frequency of selection and discrimination. A distractor can be defined as *functional* when it is intended to be plausible for those students with low achievement. For this reason, a distractor is expected to have negative discrimination and to be selected by at least 5% of the participants (Haladyna and Downing, 1988; Rodriguez, 2005; Tarrant et al., 2009; DiBattista and Kurzawa, 2011; Hingorjo and Jaleel, 2012; Gajjar et al., 2014). Distractor discrimination is usually evaluated with point-biserial correlations, which are correlations between the overall test score and a dichotomous variable (choosing/not choosing the distractor). In 2000, Attali and Fraenkel proposed a modified version in which the dichotomous variable contrasts the students who choose the distractor with those who choose the correct option (Attali and Fraenkel, 2000; Gierl et al., 2017). The analysis of distractors could also be performed alongside estimation of student ability and item difficulty, referring to specific item response theory models, i.e., Bock’s nominal-response model and Samejima’s graded response model (Gierl et al., 2017).

Among the MCIs, it is possible to find “None of the above” (NOTA) as a distractor or correct answer. This option is sometimes included among alternatives to reduce the opportunities for guessing, but the literature is discordant about its use. Item-writing guides suggested avoiding this response option (Haladyna and Rodriguez, 2013; Oermann and Gaberson, 2013), the work of DiBattista et al. (2014) revealed that using NOTA as a distractor does not change item difficulty compared with that of standard-format items that do not include NOTA. Moreover, Rodriguez (2011) and Caldwell and Pate (2013) showed that items containing NOTA as the correct alternative increased item difficulty but not discrimination power.

As reported in several studies, the number of alternatives can also be related to the quality of items and distractors. The item-writing guidelines provide suggestions about the number and type of alternatives to use in MC items (Haladyna et al., 2002; Haladyna and Rodriguez, 2013), even if there is not a general consensus in the literature. Some authors suggested producing as many plausible distractors as possible (Haladyna et al., 2002), whereas others argued that three is the optimal number of options for an item (Haladyna and Downing, 1993; Rodriguez, 2005; Vyas and Supe, 2008; Tarrant et al., 2009; Baghaei and Amrahi, 2011). As reported in Rodriguez (2005), the 3-option rule was also supported by theoretical work connecting the number of options to test efficiency and discrimination power (Tversky, 1964; Lord, 1977; Bruno and Dirkzwager, 1995). Furthermore, a meta-analysis by Vyas and Supe (2008) showed that the 3-option test does not have any significant advantage/disadvantage in its psychometric properties over 4- and 5-option tests. Generally, researchers who supported the 3-option format argued that developing many response options increases the testing time and is energy- and time-consuming for the authors.

Reducing item options can affect item quality indexes, but the directionality remains unknown. Baghaei and Amrahi (2011) reported that the number of options is related to the discrimination of alternatives and not to the difficulty of the item. In this research, the discrimination of distractors increased with the reduction in the number of alternatives. Instead, the results from Nwadinigwe and Naibi (2013) and the meta-analysis by Rodriguez (2005) showed that a decrease in the number of options increased item discrimination but reduced item difficulty. Finally, Tarrant et al. (2009) found that having fewer options decreased both item difficulty and item discrimination.

Several educational studies have compared cognitive levels and item quality, usually using Bloom’s taxonomy and some measures of difficulty and item discrimination. In 2013, Tan and Othman classified each item into three categories, combining several levels of Bloom’s taxonomy, and did not find a very strong relation with item difficulty. Moreover, Kibble and Johnson (2011) reported that no relation existed between item cognitive level and either item difficulty or item discrimination. Conversely, Kim et al. (2012) highlighted that Application and Synthesis had stronger discrimination power than did Knowledge and Comprehension, probably because the former require more critical-thinking skills. Furthermore, they found that the Analysis and Synthesis/Evaluation levels, which need a higher mastery of knowledge, were more difficult than the other categories. As mentioned above, several studies addressed the functionality of distractors in connection with structural aspects of items, such as the number of alternatives, while little attention has been paid to the relationship between the performance of distractors and the complexity of the cognitive processes underlying choosing the correct answer, that is, the cognitive level of the item.

The aim of this study is to investigate the relationship between the complexity of the items’ cognitive processes and distractor efficiency in a large item pool for a test in a statistics course. In particular, we hypothesized that items at higher levels of Bloom’s taxonomy would allow the formulation of a higher number of efficient distractors. As reviewed by Gierl et al. (2017), one of the writing guidelines for developing distractors suggests incorporating common errors into the distractors. It can be expected that student errors and misconceptions increase in number moving from the task of remembering a definition, rule or fact (knowledge level) to the task of applying knowledge and understanding to a new context (comprehension level) and to solving a problem (application level). For example, in the context of a statistics examination, an item that requires calculating a standard deviation from group data could lead to errors due to confusion between similar concepts (i.e., standard deviation, variance, sum of squares) and confusion between the number of distinct values of the variable and the number of observations. Several incorrect alternatives can be based on these common types of error.

To the best of our knowledge, no similar works are available in the literature.

## Materials and Methods

### Participants

The sample was composed of 848 undergraduate psychology students (662 women, 78.1%, and 186 men, 21.9%) enrolled at the University of Turin. Participants, aged from 18 to 64 years (*M* = 23.4, *SD* = 5.4), took the statistics test between May 2012 and February 2015. For students who failed and retook the test, only the first administration was considered.

### Materials

The study involved 174 multiple-choice items about statistics developed by professors of quantitative research methods in 2012 and checked and revised by two of the authors (ST, RR). During the statistics exam, each student received 30 items randomly selected from the item pool by computer (simple random sampling). Each item was administered to a number of students ranging from 120 to 185 (*M* = 145.0, *SD* = 12.4). No penalty was assigned for incorrect answers: a correct answer was scored as “1,” and incorrect or missing answers were scored as “0.” The score of the test ranged from 2 to 30, with a mean of 16.0 (*SD* = 5.1).

Ten items were not included in the distractors analysis because they had been modified while conducting this study. Thus, distractor analysis was performed on 164 items and 635 distractors: 79 questions (48.2%) with 300 distractors (47.2%) about descriptive statistics, and 85 questions (51.8%) with 335 distractors (52.8%) about inferential statistics. Eighty-seven percent of the items (*N* = 143) had five options, the remaining (*N* = 21) had four options. These last items were more frequent in descriptive items (16 out of 21) than in inferential items [χ^2^(1) = 7.57, *p* < 0.01]. Only 42 items (25.6%) had the NOTA alternative, and its presence was homogeneous across descriptive and inferential content and for items with 4 and 5 options.

### Data Analysis

Considering the content of the items and the goal of the examination, the categories of Knowledge, Comprehension, and Application of Bloom’s taxonomy were used to code the items. Two of the authors (ST, AT) classified the statistics item pool independently using the description of Bloom’s levels in case of statistics items reported in Dunham (2015). According to the types of tasks and the verbs used in the text for each question, each statistics item was classified into Knowledge, Comprehension, or Application categories (**Table 1** provides some examples of verbs and tasks for each category). Cohen’s Kappa was calculated to evaluate the agreement between the two judges (Kappa = 0.67, *p* < 0.001), and it was considered acceptable according to the cut-off of 0.60 in the literature (Fleiss et al., 1981; Zawacki-Richter et al., 2009). Disagreements were discussed by the two coders and resolved by consensus. Overall, 84 items (51.2%) were in the Knowledge category, 34 (20.7%) in the Comprehension category, and 46 (28.1%) in the Application category (examples of items are reported in **Appendix 1**).

**Table 1:** Bloom’s taxonomy levels with examples of descriptive verbs and tasks to be found in the statistics item pool.

Cognitive level process	General descriptors
Knowledge	Retrieving, recognizing, and recalling relevant knowledge from long-term memory. *Verbs and tasks*: recall, memorize, re-tell, repeat a definition, repeat a previously seen example, recall or identify a formula.
Comprehension	Understand uses and implications of terms, facts, methods. *Verbs and tasks*: identify an example of something, recognize a definition in an alternative wording, and describe the key features.
Application	Carrying out or using a procedure through executing or implementing. *Verbs and tasks*: use a previously seen method to compute a value or draw a generic conclusion from data. Make use of, apply practice theory, solve problems, use information in new situations.


**Adapted from Dunham (2015)*.*

Bivariate relations between Bloom’s categories and the other item features were evaluated by χ^2^ for categorical variables and one-way ANOVA for quantitative variables.

### Item Pool Evaluation

To assess the psychometric quality of the item pool, the Rasch model was applied using Winsteps (Linacre, 2012). Principal Component Analysis (PCA) of model residuals (i.e., the differences between the responses and the predicted values according to the Rasch model) was used to check the unidimensionality assumption. The reliability index (RI) was used to evaluate the reliability of the item pool, and Infit and Outfit statistics were used to assess item conformity to the Rasch model. As a rule of thumb, the following fit thresholds were considered: an eigenvalue ≤ 2 on the first PCA component and the presence of loadings <|0.38| on the first component, RI ≥ 0.70 and Infit and Outfit in the range of 0.7–1.3 (Smith, 2002; Liu, 2010; Pensavalle and Solinas, 2013).

The adequacy of the pool in terms of difficulty and discrimination was evaluated on the basis of the *P*-value (a measure of item facility), where the recommended range is 30–70 (De Champlain, 2010; Oermann and Gaberson, 2013) and on the basis of the r-PB, where the following cut-offs were used: >0.40 (very good), 0.30–0.39 (reasonably good), 0.20–0.29 (marginally good, in need of improvement), and ≤0.19 (the item must be rejected or improved by revision) (Matlock-Hetzel, 1997; Taib and Yusoff, 2014).

### Distractor Efficiency and Its Relation With Bloom’s Taxonomy

A functional distractor was defined as one that exhibited negative discrimination and was selected by at least 5% of the participants. Items might have none or only one distractor with a choice frequency ≥5% just because they are very easy (for example, with a *P*-value of 0.95, at best only one distractor could exceed the cut-off). In order not to penalize this type of item, the expected percentage of choices was calculated assuming that the choices were uniformly distributed: *q* = (100 -*P*-value)/*k*, where *k* is the number of incorrect alternatives. Among the items with a frequency <5%, those with frequency ≥*q* were classified as exceeded. Discrimination was evaluated with the traditional point-biserial correlation (r-PB) and with the modified version of the point-biserial correlation (r-PB_DC_), introduced by Attali and Fraenkel (2000):

$$r-PB=\frac{M_{D}-M}{S}\sqrt{\frac{P_{D}}{1-P_{D}}}$$

$$r-{PB}_{DC}=\frac{M_{D}-M_{DC}}{S_{DC}}\sqrt{\frac{P_{D}}{P_{C}}}$$

In the above expressions, *M* and *S* are the mean and the standard deviation of the test score on the whole sample, respectively. *M*_DC_ and *S*_DC_ are the mean and the standard deviation of the subsample who chose the distractor or the correct alternative, respectively, *M*_D_ is the mean of the examinee who chose the distractor, *P*_D_ is the proportion of students who chose the distractor and *P_C_* is the proportion of students who chose the correct option.

In r-PB_DC_ analysis, examinees who selected the distractor (D) are compared only to those who selected the correct option (C), excluding the students who selected another incorrect option from the computation. According to Attali and Fraenkel, this modified version protects against type II error, i.e., from incorrectly rejecting a distractor whose *M*_D_ is lower than *M*_DC_, but not lower than *M*.

For each item, two measures of distractor efficiency were considered (DE1 and DE2). At the item level, DE1 was defined as the percentage of distractors with a frequency ≥ 5% and an r-PB < 0. DE2 was defined as the proportion of distractors with a frequency ≥ 5% and an r-PB_DC_ < 0. As an example, let’s consider an item with three distractors (A,B,C), with the following frequency of choice and point-biserial correlations:

**Table:** 

	Frequency	r-PB	r-PB_DC_

A	7%	-0.22	-0.28
B	12%	0.05	-0.15
C	3%	-0.20	-0.24


In this example, only distractor A is efficient in terms of the DE1 index. Distractor B is inefficient because, albeit having a frequency of choice of >5%, it has an r-PB value > 0, and distractor C is inefficient because the frequency of choice is <5%. Using DE1, the score is 33% (1/3). For DE2, both distractor A and distractor B are efficient, and only distractor C is inefficient: the item score is 66% (2/3).

Both DE1 and DE2 measures could show only a few distinct values (0, 25, 33, 50, 60, 75, and 100%), and for this reason, they were recoded into dichotomous variables DE1r and DE2r (1 = percentage of functioning distractors above 50%, 0 = percentage of functioning distractors equal or below 50%). The relations among DE1r and DE2r and other item attributes were evaluated by two logistic regression models, in which DE1r and DE2r were used, in turn, as dependent variables. The independent variables were two dummy variables referring to the cognitive level, Comprehension and Application (Knowledge was used as the reference category), and the following control variables: item facility (*P*-value), item discrimination (r-PB), item content (inferential vs. descriptive), number of item options (5 vs. 4) and presence of NOTA. To assess the overall model fit, Nagelkerke’s *R*^2^ was used.

SPSS 21 was used for all analyses, with the exception of the Rasch analysis.

## Results

### Item Pool Evaluation

Overall, the Rasch results were satisfactory. The PCA of model residuals revealed that one dimension could be sufficient to account for item responses. Even though the first eigenvalue (2.1) slightly exceeded the cut-off value of 2, all of the loadings on the first component were <| 0.38|. The reliability index was over the threshold of 0.70 (RI = 0.76), and Infit and Outfit statistics were good. Few items (1 on Infit and 15 on Outfit) showed values out of the range 0.7–1.3. Moreover, item difficulties covered the range of students’ ability (**Appendix 2**, **Figure A1**). Item discrimination was very good (r-PB ≥ 0.40) in 40.2% of cases, and only 21 items (12.8%) showed very poor values (r-PB < 0.20). In terms of *P*-value, the majority of the items (68.9%) were within the established threshold of 30–70 and the mean value was 54.8 (*SD* = 18.6). The distributions of *P*-value and item discrimination are reported in **Appendix 2**, **Figures A2A,B**.

### Distractor Efficiency and Its Relation With Bloom’s Taxonomy

**FIGURE A3:**
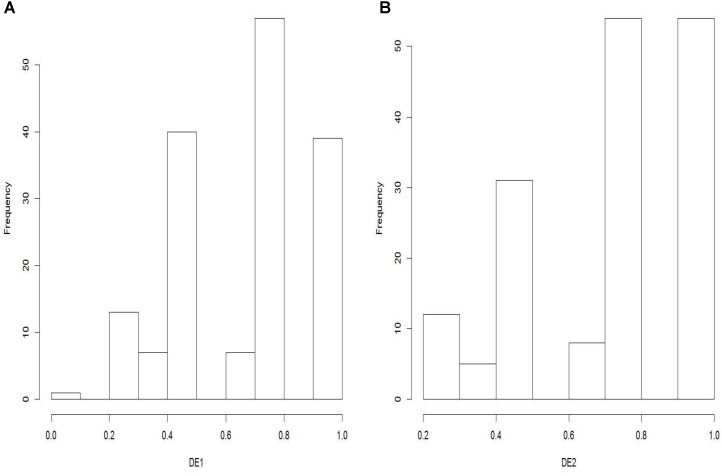
**(A)** Histogram of the percentage of efficient distractors using r-PB (DE1). **(B)** Histogram of the percentage of efficient distractors using r-PBDC (DE2).

Distractor performance was good: 74.6% of distractors had a choice frequency ≥ 5%, and most of them had a negative r-PB (88.3%). The percentage of distractors with negative discrimination rose to 95.3% when the Attali and Fraenkel r-PB_DC_ was employed. Nearly 70% of distractors were functional: using r-PB as the discrimination measure, 68.5% of the distractors were functional, and a slightly higher percentage of items, 73.2%, were functional when r-PB_DC_ was used (**Table 2**). **Appendix 3** shows the distributions of frequency of choice and point-biserial correlations for the 635 distractors (**Appendix 3**, **Figures A1**, **A2A,B**) and the percentage of efficient distractors at the item level (DE1 and DE2) in **Appendix 3**, **Figures A3A,B**.

**Table 2:** Item distractor performance (*n* = 635).

	*N* (%)
Frequency ≥ 5%	474 (74.6)
r-PB < 0	561 (88.3)
r-PB_DC_ < 0	605 (95.3)
Frequency ≥ 5% and r-PB < 0	435 (68.5)
Frequency ≥ 5% and r-PB_DC_ < 0	465 (73.2)


*r-PB, discrimination computed with point-biserial correlations; r-PB_*DC*,_ discrimination computed with the Attali and Fraenkel measure.*

**Table 3** shows the main bivariate results at the item level. Knowledge items were quite equally represented in descriptive and inferential topics (58.3% inferential), whereas Comprehension was under-represented (17.6%) and Application was over-represented (65.2%) among inferential items (*p* < 0.001). The majority of items with NOTA were in the Knowledge group (*p* = 0.003). No association was found between cognitive demand classification and either the number of options (*p* = 0.979) or item discrimination (*p* = 0.891). On average, item facility (*P*-value) was greater for those items classified as Knowledge (57.1) or Comprehension (59.9) than for those classified as Application (46.8). Both DE1 and DE2 indicators were significantly related to item cognitive level (*p* = 0.013, *p* = 0.001, respectively). According to Bonferroni *post hoc* analysis, only the difference between Knowledge (*M* = 64.9) and Application (*M* = 77.0) was statistically significant when DE1 was used, whereas the average of DE2 for both Knowledge (*M* = 69.1) and Comprehension (*M* = 68.1) was statistically lower than that of Application (*M* = 84.4). The effect size was negligible in the former case (η^2^ = 0.013) and small in the latter (η^2^ = 0.024). An analogous pattern of results was obtained using the dichotomized version of distractor efficiency measures, DE1r and DE2r. The percentage of items with more than 50% functioning distractors was greater in the Application group (76.1 and 89.1%, respectively, for DE1r and DE2r) than in the others two groups. The association was statistically significant only for DE2r (*p* < 0.01), and only the effect size for DE2r was not negligible (Phi = 0.18 for DE1r and Phi = 0.25 for DE2r).

**Table 3:** Association between Bloom’s classification and the other item attributes.

	Knowledge *n* = 84	Comprehension *n* = 34	Application *n* = 46	*P*
Inferential^a^	49 (58.3)	6 (17.6)	30 (65.2)	<0.001
Nota^a^	31 (36.9)	4 (11.8)	7 (15.2)	0.003
5-options^a^	73 (86.9)	30 (88.2)	40 (87.0)	0.979
Item r-PB^b^	0.35 (0.1)	0.36 (0.1)	0.36 (0.1)	0.891
*P*-value^b^	57.1^c^ (20.3)	59.9^c^ (15.3)	46.8^d^ (14.9)	0.002
DE1^b^	64.9^c^ (23.6)	65.0 (25.4)	77.0^d^ (21.3)	0.013
DE2^b^	69.1^c^ (24.4)	68.1^c^ (23.6)	84.4^d^ (23.7)	0.001
DE1r = 1^a^	50 (59.5)	18 (52.9)	35 (76.1)	0.071
DE2r = 1^a^	54 (64.3)	21 (61.8)	41 (89.1)	<0.005


*(a) number of items and columns percentages (in brackets); P in the last column is the p-value associated with the χ^2^ statistics; (b) means and standard deviations (in brackets); P in the last column is the p-value associated with the one-way ANOVA; different letters (c and d) mean that the difference was statistically significant in the Bonferroni post hoc analysis.*

Based on the number of functioning distractors per item, two dichotomous measures of distractor efficiency (DE1r, related to r-PB, and DE2r, related to the Attali and Fraenkel measure) were computed and used as dependent variables in a logistic regression model. In both DE1r and DE2r, a value of 1 means that more than 50% of the distractors were functional. The percentage of items with a distractor efficiency measuring 1 was as follows: 68.3% (DE1r) and 73.2% (DE2r).

As shown in **Table 4**, in both of the regression models, item facility (*P*-value) and the item discrimination index (r-PB) showed a significant relation with distractor efficiency. Specifically, distractor efficiency was greater when item discrimination and item difficulty were higher. A significant relation with the number of options emerged only when the Attali and Fraenkel index was employed. In this case, distractor efficiency decreased when moving from 4 to 5 response options.

**Table 4:** Logistic regression estimates (*n* = 164) with distractors efficiency as dependent variable (DE1r, DE2r) and item attributes as independent variables.

	DE1r			DE2r		
		
	*B*	Exp(*B*)	Sig.	*B*	Exp(*B*)	Sig.
Constant	0.71	2.04	0.482	4.64	104.10	0.001
*P*-value	-0.04	0.96	0.001	-0.07	0.93	<0.001
Item PB correlation	0.79	2.20	<0.001	0.65	1.91	0.001
Number of options (5 vs. 4)	-1.11	0.33	0.071	-1.97	0.14	0.011
NOTA	0.50	1.65	0.281	0.31	1.36	0.568
Inferential vs. descriptive	-0.03	0.98	0.950	-0.52	0.60	0.288
Bloom’s comprehension	-0.21	0.81	0.663	-0.21	0.81	0.706
Bloom’s application	0.54	1.71	0.261	1.08	2.93	0.079


**Nagelkerke R^*2*^ = 0.28 (DE1r) and 0.41 (DE2r). DE1r, distractor efficiency measured with the discrimination computed with the PB correlation; DE2r, distractor efficiency measured with Attali and Fraenkel’s discrimination measure; PB, point-biserial.**

When controlling for the other items attributes, the relation with the item cognitive level was not statistically significant anymore. However, regression coefficients were of the right sign, and they were large, especially that of the DE2r model [Exp(*B*) = 2.93]. As Application level was associated with *P*-value (**Table 3**), and *P*-value was related to distractor efficiency (Pearson correlations were *r* = -0.26 for DE1r and *r* = -0.44 for DE2r), it was suspected that there was a confounding effect and a further regression analysis without *P*-value was conducted. In this analysis (results are not shown), the coefficient for Application was statistically significant in both DE1r and DE2r models [DE1r: Exp(*B*) = 2.50, *p* < 0.05; DE2r: Exp(*B*) = 5.15, *p* < 0.01].

Overall, the pattern of relationships was likely the same across the two efficiency indicators, but the overall fit of the model was better when r-PB_DC_ was used to define efficiency (Nagelkerke *R*^2^ = 0.41 vs. 0.28). The *R*^2^ increment was mainly due to the stronger influence of *P*-value and item point-biserial correlation.

## Discussion

The aim of this study was to investigate whether distractor quality was related to the type of mental processes involved in answering MCIs. In particular, it was hypothesized that higher levels of cognitive processing enable test constructors to produce more functioning distractors. To assess this hypothesis, an item pool for a statistics examination was analyzed. The pool showed acceptable reliability, a satisfactory spread of item difficulty, and only few items that did not fit the Rasch model. Moreover, approximately 40% of the items had very good discrimination, and approximately 70% of the distractors properly functioned. These results are in line with (or better than) those of previous classroom test research studies considering that in the present study, the vast majority of items had five options, whereas in previous studies, four options was more typical (Tarrant et al., 2009; DiBattista and Kurzawa, 2011; Hingorjo and Jaleel, 2012; Gajjar et al., 2014).

The bivariate results mainly supported the authors’ hypothesis that distractor efficiency was related to Bloom’s cognitive processing categories. Specifically, items classified at the Application level had a great number of efficient distractors compared to items at the Knowledge level and by using the Attali and Fraenkel discrimination index, the mean efficiency of Application items was also higher than that of Comprehension items. The relation lost statistical significance in the regression models. There are two possible reasons. First, the effect size was small and requires a larger sample of items. Second, some confounding effects between cognitive levels and *P*-value (the opposite of item difficulty) could have been at work because, according to the bivariate results, Application items were more difficult than the others, and the *P*-value, in turn, was a strong predictor of distractor efficiency.

The current findings could be interpreted in light of cognitive diagnostic models that have been proposed to formulate and analyze distractors (Gierl et al., 2017). According to this approach, distractors can be derived from the different stages of understanding that students need to master in order to answer the MCI correctly or from the different attributes (knowledge, skill or cognitive process) needed to choose the correct response option. When the item requires a calculation or solving a problem (Application level), typically, more stages of understanding and/or more attributes are involved than those involved in a Knowledge item. For this reason, it could be easier to formulate a larger number of functioning distractors. When test developers decide to use the same number of options for all items regardless of the complexity of the cognitive task, the distractor efficiency can be lower for Knowledge items than for Application items.

Some other results deserve to be mentioned. In the present study, a significant relation was observed between distractor efficiency and both item difficulty and discrimination. These results agree with previous findings (Haladyna and Downing, 1988; Tarrant et al., 2009; Hingorjo and Jaleel, 2012), and they partially agree with Haladyna and Downing (1993), who found that the number of effective distractors was related to discrimination but unrelated to difficulty. Moreover, the Attali and Fraenkel point-biserial correlation seems an appropriate discrimination index. As the authors highlighted and found in the present study, this measure leads to a more favorable evaluation, reducing the number of non-functioning distractors. Further evidence of its adequacy came from our regression results. When the dependent variable was based on the Attali and Fraenkel index, *R*^2^ was appreciably greater than the value obtained when the dependent variable was based on the traditional index. In particular, the two independent variables logically related to distractor efficiency, item difficulty and discrimination, improved the strength of the relationship.

This study is limited by its sample size. The number of examinees per item was small, and each item, on average, was administered to a sample of 145 students. A larger sample of examinees could improve the evaluation of item and distractor performance. Additionally, the item pool needs to be enlarged to ensure that small effects, such as those related to Bloom’s categories, could robustly emerge. Moreover, a more balanced item pool in term of item difficulty across the three Bloom levels is necessary to disentangle the relation between distractor efficiency, difficulty and the complexity of the cognitive task.

The present findings are encouraging and offer suggestions for further research. According to the literature (Haladyna and Downing, 1988; Rodriguez, 2005; DiBattista and Kurzawa, 2011), in the present study, a negative distractor discrimination was required, but this rule might penalize those distractors that are attractive for high achievers (Levine and Drasgow, 1983). Further research could investigate how the relationship with cognitive level might change when analyses are performed with a distinction between incorrect options that attract high, middle or low achievers. Moreover, further studies with a larger sample could deepen the evaluation of dimensionality, comparing the unidimensional model with a multidimensional Rasch model, in which each latent variable corresponds to one of the different cognitive levels. In fact, in the present study, the eigenvalue criteria of 2 was slightly exceeded, which could signal the presence of marginal multidimensionality, most likely connected to the distinction between the cognitive requirements to provide the correct answer. A further line of research could employ explanatory item response modeling (EIRM, Wilson et al., 2008) to simultaneously estimate item and person latent scores and assess the influence of item features and participant characteristics on the parameter estimates. This approach would allow a better comprehension of the relationship between cognitive complexity levels and item difficulty in light of the not always convergent results reported in the literature (Kibble and Johnson, 2011; Kim et al., 2012; Tan and Othman, 2013).

## Conclusion

There seems to be a relation between item cognitive level and distractor efficiency. The direction of this relation is the expected one, with Application items having more efficient distractors than Knowledge items. Given the heterogeneity of the results reported in the literature, further studies on the performance of distractors should be encouraged.

## Ethics Statement

Ethical approval was not sought for this secondary analysis, which was based on anonymous data from the archive of statistics written examinations. Students gave their written consent by filling-in an anagraphic form for the examination.

## Author Contributions

ST and RR conceived the study. ST and AT did the analyses. ST, AT, and RR wrote the paper. All authors discussed the results together and contributed to the final manuscript, doing critical revisions and giving suggestions. All authors read the manuscript and approved the submitted version.

## Conflict of Interest Statement

The authors declare that the research was conducted in the absence of any commercial or financial relationships that could be construed as a potential conflict of interest.

## Figures and Tables

**FIGURE A1:**
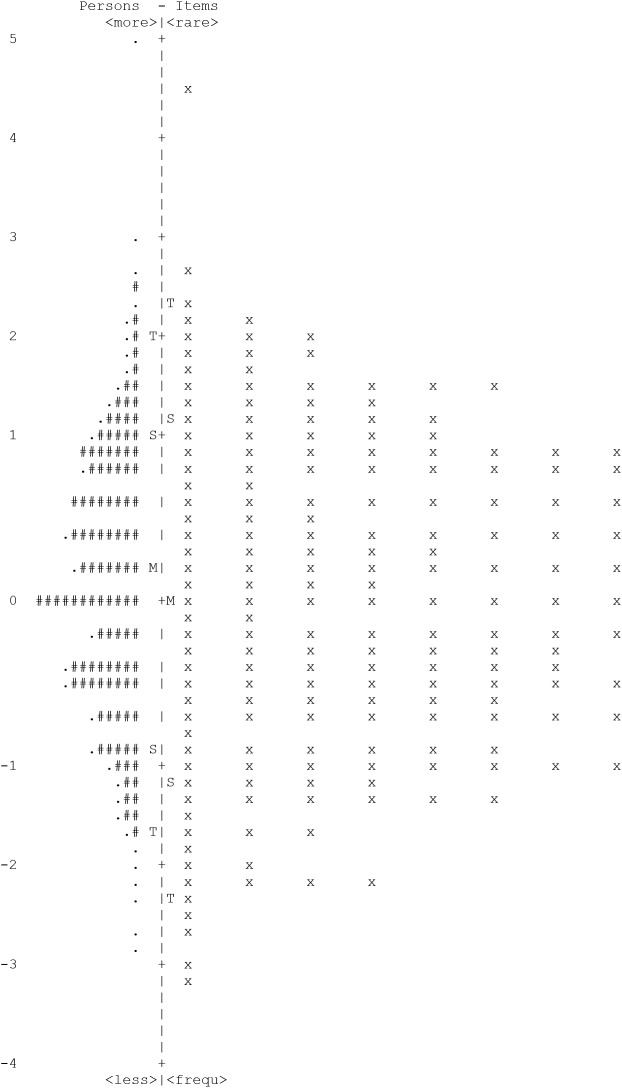
Rasch analysis: item-person map. Each ‘#’ is 7 persons; each ‘x’ is an item.

**FIGURE A2:**
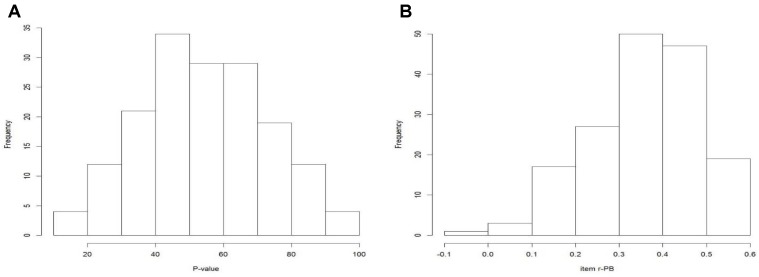
**(A)** Histogram of item facility (*P*-value). **(B)** Histogram of item discrimination (r_PB).

**FIGURE A1:**
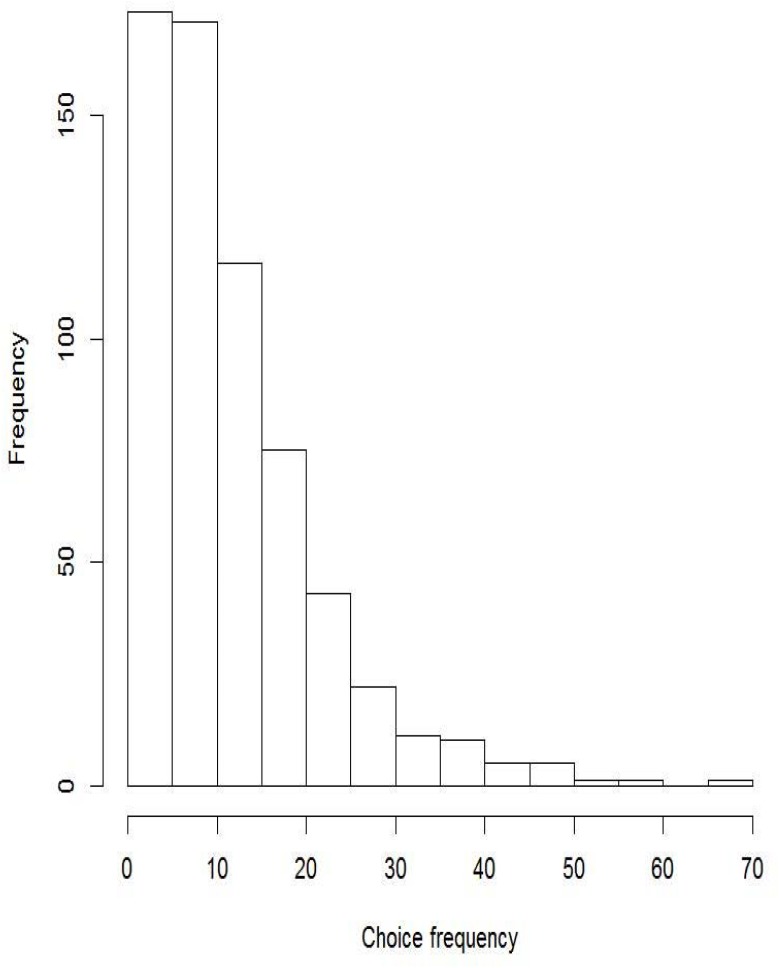
Histogram of choice frequency for each distractor.

**FIGURE A2:**
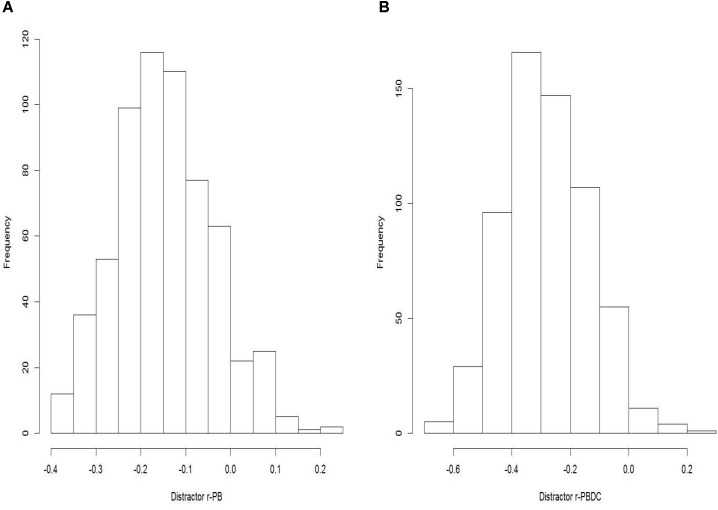
**(A)** Histogram of distractor discrimination using r-PB (distractor r-PB). **(B)** Histogram of distractor discrimination using r-PBDC (distractor r-PBDC).

## Appendix 1 – Examples of Statistics Items Classified According to Bloom’s Taxonomy

An example of a statistics item assigned to the Knowledge category is:

Which of the following graphs is used to illustrate the relation between two quantitative variables?

(A) Scatter plot
(B) Pie chart
(C) Box plot
(D) Stem and leaf plot
(E) Histogram

An example of a statistics item assigned to the Comprehension level is:

The codes shown here (1, 2, 3 at the left of the arrows) are the answer modalities of an ordinal variable. Which of the following transformations is correct?

**Table:**

(A)	(B)	(C)	(D)
1 → 2	1 → 1	1 → 2	1 → 1
2 → 3	2 → 3	2 → 2	2 → 2
3 → 6	3 → 2	3 → 3	3 → 2

An example of a statistics item assigned to the Application category is:

The measurement of the height of 40 people shows an arithmetic mean of 179.3 cm and a variance of 129.7. Calculate the sum of squares.

(A) -49.6
(B) 3.24
(C) 5188
(D) 1.38
(E) 7172
